# Low-potential immunosensor-based detection of the vascular growth factor 165 (VEGF_165_) using the nanocomposite platform of cobalt metal–organic framework

**DOI:** 10.1039/d0ra03181j

**Published:** 2020-07-21

**Authors:** Sima Singh, Arshid Numan, Yiqiang Zhan, Vijender Singh, Aftab Alam, Tran Van Hung, Nguyen Dang Nam

**Affiliations:** School of Pharmacy, Sharda University Greater Noida 201310 Uttar Pradesh India; State Key Laboratory of ASIC and System, SIST, Fudan University 200433 Shanghai China; Department of Pharmacognosy, College of Pharmacy, Prince Sattam Bin Abdulaziz University Al-Kharj Kingdom of Saudi Arabia; Institute of Research and Development, Duy Tan University Danang 550000 Vietnam tranvanhung9@duytan.edu.vn nguyendangnam@duytan.edu.vn; The Faculty of Environmental and Chemical Engineering, Duy Tan University Danang 550000 Vietnam

## Abstract

The vascular endothelial growth factor 165 (VEGF_165_) is a quintessential biomarker in cancers. An easy and precise tool for the early detection of malignancies is required for rapid care and metastasis prevention. Cobalt-based metal–organic framework (Co-BTC-GO-MOF) nanoparticles have been used as a signal carrier for the anti-VEGF_165_ signaling antibody. Cobalt-based MOF was synthesized using cobalt (Co), benzene-1,3,5-tricarboxylate (BTC), and graphene oxide (GO) applying a hydrothermal method. Structure, compositions, size and morphology of the qualified sensor are determined by using distinctive analytical techniques. The Co-MOF nanoparticles are found to be thermostable, as revealed by thermal stability assay. The strategy utilises an impedimetric and differential pulse voltammetry (DPV) techniques in the presence of the [Fe(CN)_6_]^3−/4−^ redox system. Compared to earlier results, this assay resulted in higher sensitivity with the limit of detection (LOD) found to be 5.23 pM in a 0.01 M buffer solution of pH 7.4 using linear scale voltammetry at room temperature. The resulting Co-BTC-GO-MOF immunosensor shows high responsiveness and selectivity in detecting VEGF_165_ in real-time serum samples of cancer patients. The electrochemical performance studies confirm that the intended proposed immunosensor could pave the way for the future advancement of high-performance, sensitive, reproducible and robust immunosensors for the cost-effective and initial phase detection of cancer in the future.

## Abstract 2

The vascular endothelial growth factor 165 (VEGF_165_) is a quintessential biomarker in cancers.

## Graphical abstract

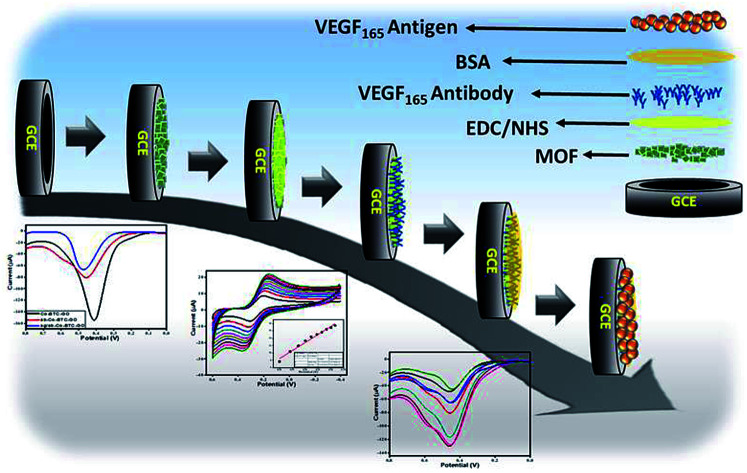

## 1 Introduction

Vascular endothelial growth factor (VEGF) is a hypoxia-inducible protein that results from alternate splicing of the VEGF gene.^1^ It performs a vital role in the development of the mammalian vascular system in adult tissues during embryogenesis and angiogenesis. An abnormal level (either over expression or down-regulation in the level of VEGF) is responsible for the induction of various diseases. The over expression represents a potent promoter in the pathophysiology of angiogenesis associated with the growth of tumors. VEGF is overexpressed in numerous human cancers like lung, breast, brain cancer, urinary tract and gastrointestinal cancers.^2^ Alternatively, when the level of VEGF is lower, it is an indication of developing degeneration of neurons by limiting the neural tissue perfusion. Different CNS disorders, such as Parkinson's disease and brain injuries, are associated with the down-regulation of VEGF.^3^ Some major expressed variants of VEGF isoforms from a single VEGF gene consist of 121, 145, 165, 183, 189 and 206 amino acids. Out of these six isoforms, VEGF_165_ is the most predominant VEGF-A isoform, which is over expressed in tumor cells during preliminary tumor growth phase, especially, in breast and lung cancers and possible predictor of cancers. Therefore, VEGF_165_ is an important predictive biomarker for early detection of cancer in healthcare settings. The determination of the level of VEGF_165_ in blood, irrespective of their type, can be a fruitful approach to clinical diagnosis of cancer.^4^

Till date, there are several methods have been reported for detection and quantification of VEGF_165_, which include enzyme-linked immunosorbent assay (ELISA),^5^ optical methods,^6^ electrochemical immunoassay methods^7^ and radioimmunoassay techniques.^8^ The ELISA continues to be the gold standard for clinical quantification of many protein biomarkers with excellent specificity and very low limits of detection (LOD).^9^ However, the requirement of sophisticated instrumentations, complex detection protocols with a long testing time, lack of portability, difficulty with multiplexed sensing and high cost, it is not suitable for point-of-care applications.^10^ To overcome these shortcomings of the existing detection techniques, the emerging electrochemical immunosensor has inherent benefits. As compared to traditional molecular recognition system selector chemical immunosensor is widely accepted because of its simple instrumentation, high sensitivity, affinity, portability, rapidity, reproducibility and wide applicability at an economical price. They are also regarded as important tools for monitoring and preventing various diseases, including cancers. On the other hand, due to the high specificity of the antibody towards specific antigen and the faster reaction time, the antibody can be explored as a potential tool in detecting substances.^11^ In consequence, it is highly desirable to design a novel nanomaterial for selective and sensitive detection of VEGF_165_ even at lower concentrations.

In the past decade, metal–organic frameworks (MOFs) based nanomaterials have emerged as an excellent alternative tool for quantification of biomarkers. It has been reported to be a very useful tool to modify electrodes due to its highly selective reactivity, high yield, moderate reaction conditions and simplicity. On top of that, the electrochemical application of MOFs in recent years has been fascinated due to its large pore size, high specific surface area, and good conductivity. Additionally, the specific antibodies can be incorporated in the architecture of MOFs by covalent bonding.^12^ Consequently, MOFs can serve as an excellent platform to effectively ligate the recognized element to the surface of the electrode, which leads to adequate signal amplification.

In the present work, nano-porous MOF is designed. There are several electrochemically active MOFs have been introduced as an electrocatalyst, electroactive signal probes or redox-active species, such as Co-MOF, Ni-MOF and Cu-MOF, for detection of various analytes.^13^ However, the low dispersibility, poor electronic conductivity and limited sensitivity of MOFs is still a challenge.^14^ The addition of graphene oxide (GO) can mitigate these problems due to its unique structure and distinguished physiochemical and mechanical properties. It can produce a synergetic effect by increasing the dispersibility, subsequently electronic conductivity and sensitivity of the composite.^15^ Based on these advantages, in the present work, for the first time, cobalt (Co), benzene-1,3,5-tricarboxylate (BTC) and GO-based MOFs (Co-BTC-GO-MOFs) were synthesized by a simple hydrothermal method for detection of VEGF_165_ on both, signal on impedimetric and differential pulse voltammetry (DPV) techniques. The use of CO-BTC-MOFs combined with GO yielded improved sensitivity and stability. Moreover, the signal readout was also much simpler and low-cost with the target to provide good sensing performance, such as fast reaction, high sensitivity, with good stability and reproducibility.

## 2 Materials and methods

Human recombinant VEGF_165_, anti-VEGF_165_ antibody, *N*-hydroxysuccinimide (NHS), 1-ethyl-3-(3-dimethylaminopropyl)carbodiimide hydrochloride (EDC), dimethylformamide (DMF) and ethanol (95%) were purchased from Sigma-Aldrich, India. Just to highlight that anti-VEGF antibody and VEGF_165_ were stored at 4 °C and −20 °C, respectively, before use. Freshly aqueous solutions of EDC and NHS were prepared before using. All solutions were prepared using deionized water (DI) from a Millipore system. Cobaltous chloride, potassium ferricyanide, 1,3,5-benzenetricarboxylic acid (BTC) and GO were purchased from Merck. Sodium dihydrogen orthophosphate dehydrate (Merck) and sodium phosphate dibasic dehydrate (Sigma-Aldrich) were used to prepare a 0.1 M phosphate buffer of different pH to carry out pH study. All chemicals were of analytical grade and used without any further purification.

### 2.1 Apparatus

Electrochemical measurements were performed on a three-electrode cell system of the electrochemical workstation (multi-channel Auto-lab, PGSTAT30 USA) using glassy carbon electrode (GCE – 3 mm diameter) as working, Ag/AgCl, as reference and platinum wire as a counter electrode, cyclic voltammetry (CV) and differential pulse voltammetry (DPV) was performed in a potential window of −0.4 to 0.6 V in 0.1 M PBS at room temperature. Morphology and surface of the Co-BTC-GO-MOFs was observed under the field emission-scanning electron microscope (FE-SEM; JEOL JSM-7600F, Japan). The Co-BTC-GO-MOFs were sputter-coated with gold to avoid accumulation of charges before FE-SEM examination. For texture analysis and imaging the sample holder was then placed into the FE-SEM. Observation magnification may be regulated and considered based on the requirements. Further, high resolution-transmission electron microscopy (Hitachi HT7700, Japan) were used for the determination of size. It is also helpful in providing direct images of the atomic structure of the Co-BTC-GO-MOFs. The Co-BTC-GO-MOFs were dissolved in DI water then mounted onto the surface of a copper grid. The excessive sample was removed by blotting off with filter paper, then dried at room temperature before analysis. X-ray diffraction patterns (XRD), were recorded using Philips X'Pert, UK fitted with Cu-K_α_ X-ray radiation (*λ* = 1.5418 Å) at a scan rate of 0.02 s^−1^, which is operated at 40 kV and 50 mA. Fourier transform infrared spectroscopy (FTIR, Thermo Scientific Nicolet ISIO Smart ITR, USA) and thermal stability of the prepared samples were recorded using Q Series Thermal Analyzer DSC/TGA (Q600). The Brunauer–Emmett–Teller (BET) specific surface area and porosity were recorded using Micromeritics Tristar II ASAP 2020 system, USA surface area and porosity analyser.

### 2.2 Experimental procedure

#### Preparation of Co-BTC-GO-MOFs by single-step hydrothermal autoclave

Co-BTC-GO-MOFs were synthesized by mixing 0.535 mg of cobaltous chloride (2.25 mmol) dissolved in 7.5 mL of DI water and 0.264 g of BTC (0.264 mmol) dissolved in 7.5 mL of ethanol. The mixture was kept under constant magnetic stirring for 10 min. A 2 mg of GO was added in the above mixture under constant stirring, and the whole mixture is in Teflon lined stainless steel autoclave (200 mL), which is subjected to hydrothermal reaction at 100 °C for 24 hours. The precipitate was then washed with a copious amount of DI water to remove the excess reagents using centrifuged at 4000 rpm. The finished final product was obtained by centrifugation, washed several times with DI water and kept for vacuum drying to achieve constant weight at room temperature. Subsequently, for further use, the lyophilised sample was kept in airtight glass vials.^16^

#### Fabrication of immunosensors on Co-BTC-GO-MOFs modified GCE

Before proceeding to fabricate the developed Co-BTC-GO-MOFs, the surface of the bare GCE was electrochemically clean in 0.1 M H_2_SO_4_ solution and polished with α-alumina powder (1.0, 0.3 and 0.05 μm) on a polishing cloth to achieve a mirror like surface. Further, it was again ultrasonically cleaned in the solution mixture of DI water and ethanol. It was finally dried with acetone.

Subsequently, 1 mg mL^−1^ of dispersion of Co-BTC-GO-MOFs was prepared in DI water. In order to prepare an electrode, 5 μL dispersion mixture of Co-BTC-GO-MOFs was dropped onto the dry and clean GCE surface. It was kept for drying under an infrared lamp. Following the drying process, the electrode was then activated by the treatment of EDC/NHS (30 mM/2 mM) for 2 h. It was subsequently washed with DI water. Further, the electrode was incubated in VEGF_165_ antibody solution (10 μg mL^−1^) prepared in 0.1 M PBS, pH 7.4. It was immersed for 4 h. Finally, the modified electrode was washed with 0.1 M PBS and concurrently blocked with bovine serum albumin (BSA) solution (1%, 10 μL) to eliminate the non-specific binding effects. It was followed by careful washing in 10 mM PBS (pH 7.4). Thus, the fabricated immunosensor was obtained.

#### Electrochemical measurement

Once the antibody-modified sensor was obtained, different concentrations of solution (5 μL) were introduced onto the surface of the developed electrode. The desired dilution of VEGF_165_ antigen were freshly prepared in PBS by serial dilution from the stock solution. The prepared antigen solutions were kept on ice throughout the experiments. 5 μL of each antibody solution was drop casted on the biosensor and incubated at room temperature for 1 hour. After that, the sensor was rinsed with PBS to remove any unbound peptides. The measurement time for each sensor is approximately 3–4 minutes. Here, the DPV technique was adopted to measure the responsive signals from redox probes. The variable experiment parameters were set for the current measurements are initial potential: −0.8 V; final potential: 0.4 V; pulse amplitude: 0.05 V; pulse width: 0.25 s; sample interval: 0.002 s.

#### Real sample analysis

Serum sample analysis was carried out for the newly developed immunosensor to check the its practical application and feasibility. We perform the real-sample analysis in sheep blood sample. Sheep blood was obtained from Rockland Immunochemicals, Inc. Blood sample of sheep was diluted with PBS solution in the ratio 1 : 100. The desired dilutions of the VEGF_165_ antigen were prepared in serum by mixing with PBS by using serial dilution technique. Then, biosensors were incubated with spiked samples for 60 min followed by washing with PBS and electrochemical measurement were recorded.

## 3 Results and discussion

**Fig. 1:**
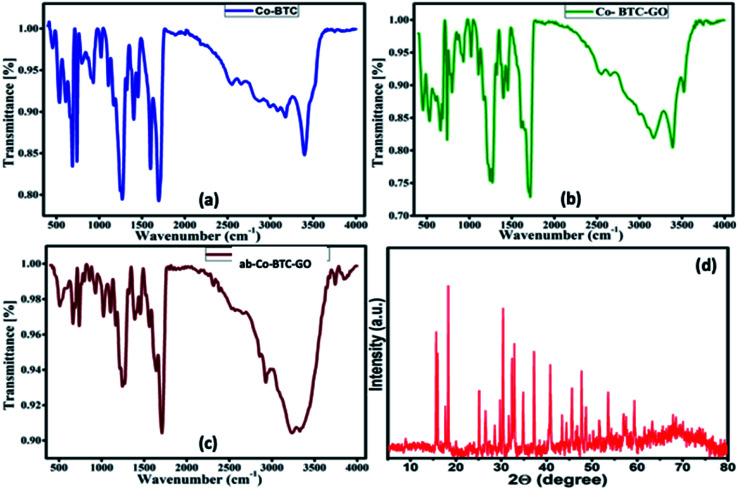
FTIR spectrum of (a) Co-BTC, (b) Co-BTC-GO-MOFs, (c) ab-Co-BTC-GO, and (d) XRD pattern of Co-BTC-GO-MOFs.

To explore the functional groups, present in the prepared MOFs, FTIR analysis was carried out. All the MOFs composites (Co-BTC, Co-BTC-GO), and antibody-Co-BTC-GO (ab-Co-BTC-GO) showed similar spectra with exceptions of emerging peaks for composites with graphene oxide and antibody or both. The sharp peak at about 3398 cm^−1^ in both Co-BTC (Fig. 1a) and Co-BTC-GO-MOFs (Fig. 1b) is attributed to free O–H bonds in the structure. The broad peak at 3327 cm^−1^ for the ab-Co-BTC-GO is attributed to intermolecularly bonded O–H as shown in Fig. 1c. The characteristic peak at 1700 cm^−1^ for all the MOFs was attributed to C=O present in the structures, while at about 1245 cm^−1^ was assigned to C–O. At the fingerprint region, the peak between 600–690 was linked to the Co–O bond.

The crystal structure of the Co-BTC-GO-MOFs was explored using XRD, as shown in Fig. 1d. The Co-BTC-GO-MOFs showed clearly sharp peaks, which is attributed to a well-defined crystal structure. The interlayer spacing was calculated from the Bragg's law, *λ* = 2*d* sin *θ*. The diffraction peak at 2*θ* = 15° (indexed 001) is attributed to the GO with an interlayer spacing of 1.38 Å, indicating the presence of GO in prepared MOFs. The other main characteristic peaks were observed at 2*θ* = 19, 30, 31, 37, 40 and 63°, which correspond to (440), (220), (100), (011), (400) and (511) crystal planes cobaltous chloride, 1,3,5-BTC, respectively. This results was in a good agreement with what has been reported in previous works.^17^

**Fig. 2:**
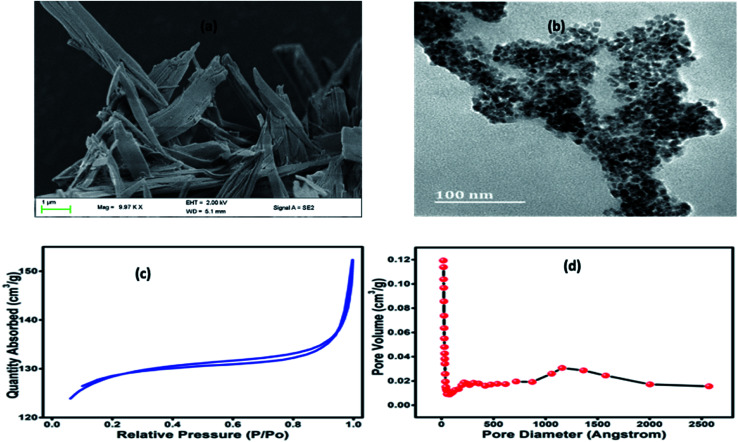
(a) FE-SEM image of Co-BTC-GO-MOFs; (b) HR-TEM image of Co-BTC-GO-MOFs; (c) BET nitrogen adsorption–desorption isotherms; (d) the BHJ pore volumes.

It can be seen from Fig. 2a that Co-BTC-GO-MOFs exhibits a stacked hexagonal phase with flake-like morphology. The thickness of the homogeneous hexagonal flake is measured to be less than 50 nm, and the width is about 1 μm. As it has been depicted in the connecting section, the higher activity of Co-BTC-GO-MOFs is a consequence of the application of the synthesized hexagonal flake-like MOFs with a lower thickness than that of the general cobalt MOFs. The results obtained are in line with reported literature on the Co-MOFs synthesized at relatively lower temperatures.^18–20^ To visualize the inner microstructure of the Co-BTC-GO-MOFs, TEM was employed. TEM images (Fig. 2b) depict the inserted co-nanoparticles inside the nanopores of the flake MOFs. Consequently, the TEM image demonstrated that Co-BTC-GO-MOFs with a size of 100 nm into the pores of MOFs.

Nitrogen adsorption isotherms for the Co-BTC-GO-MOFs were represented in (Fig. 2c and d). The Brunauer–Emmett–Teller (BET) surface area and cumulative adsorption volume of pores were found to be 409.0040 m^2^ g^−1^ and 0.052512 cm^3^ g^−1^, respectively. The adsorption isotherms curve is of type IV and adsorption–desorption H_4_ hysteresis loop, a characteristic of mesoporous materials in powder form. The large BET surface area exhibited by Co-BTC-GO-MOFs could have been due to small particles agglomerates which was imbedded on the large particles (see Fig. 2a), resulting into a microporous structure with promising catalytic activity. This finding evidences of an overestimation of its BET surface area and pore volume; hence, the surface coverage for the Co-BTC-GO-MOFs could be greater and, therefore, in better agreement with the behaviour observed by other researchers.^21^

**Fig. 3:**
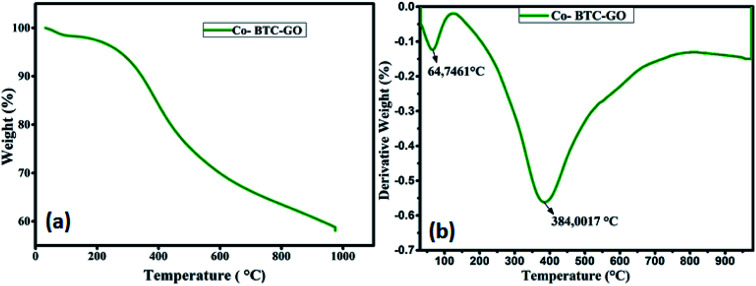
(a) TGA curves of Co-BTC-GO-MOFs measured under air and; (b) the corresponding derivative weight percent *versus* temperature curves.

The thermal stability of the formulated composite was studied using TGA analysis at the temperature range between 25–800 °C. Initial loss of weight of the Co-BTC-GO-MOFs during initial sections of TGA curves could be correlated with the evaporation of coordinated water from the complex molecule below 100 °C as shown in Fig. 3a. The gradual weight loss experienced at about 200 °C can be posited to loss of CO, CO_2_ from GO due to destruction of oxygen-containing functional group in it. Further, the Co-BTC-GO-MOFs showed a subsequent loss of weight during 300 °C to 500 °C temperature, correspond to the decomposition process of organic ligands (GO and MOFs). Thus, the second loss of weight for the composite Co-BTC-GO-MOFs was recorded at 384 °C, which resulted in a 60% loss of weight. This could further be explained by the improved thermo-stability of the developed Co-BTC-GO-MOFs as shown in Fig. 3b. At around 600 °C, the mass loss was due to combustion of carbon skeleton in organics while the residual mass of about 40% over 900 °C was attributed to the remaining metallic cobalt part.

**Fig. 4:**
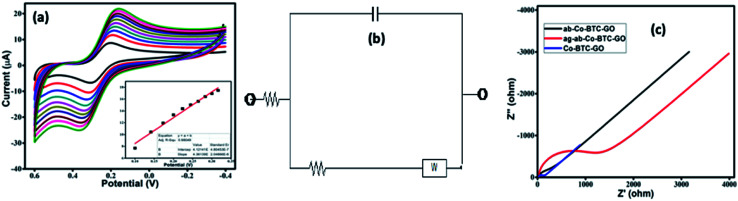
(a) Scan rate studies of Co-BTC-GO/GCE from (0.01 to 0.10 V s^−1^) carried out in 1 mM [Fe(CN)_6_]^3−/4−^ in 1 M KCl, inset – calibration plot for anodic peak current *vs.* root of scan rate; (b) equivalent circuit model; (c) EIS spectra in 1 mM [Fe(CN)_6_]^3−/4−^ in 1 M KCl at Co-BTC-GO/GCE, ab-Co-BTC-GO/GCE, ag-ab-Co-BTC-GO/GCE. Inset – Randles equivalent circuit model.

The CVs were performed to carry out the electrochemical characterization of the developed Co-BTC-GO-MOFs modified GCE. This CV curve was recorded executed in 1 mM [Fe(CN)_6_]^3−/4−^ in 1 M KCl as the electrolyte. CVs of [Fe(CN)_6_]^3−/4−^ redox couple is a suitable probe to analyze the surface properties of the electrode during numerous modification steps.^22^ Later, the electrochemical surface area of the modified electrode was calculated using the Randles–Sevcik eqn (i):i
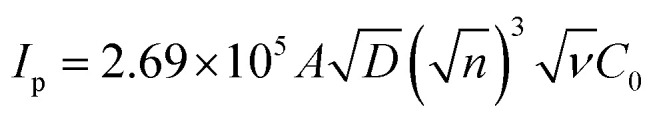
where, *I*_p_ is the peak current in ampere, *A* represents the surface area of the electrode (cm^2^), *D* is the diffusion coefficient which is 7.60 × 10^−6^ cm^2^ s^−1^ for K_3_[Fe(CN)_6_], *n* represents the number of electrons transferred, which in case of K_3_[Fe(CN)_6_] is equal to 1, *ν* is the scan rate (V s^−1^) and *C*_0_ is the concentration in mol cm^−3^. The slope of plot *I*_pa_*vs. ν*^1/2^ in Fig. 4a was used to calculate the electrochemical or active surface area. The active surface areas for GCE, Co-BTC/GCE, and Co-BTC-GO/GCE were found to be 0.03608 cm^2^ and 0.05877 cm^2^, 0.09667 cm^2^, respectively. The large active surface area of Co-BTC-GO/GCE also helps in the immobilization of a higher quantity of antibody on the modified GCE, leading to better detection of VEGF_165_.

Additionally, charge transfer kinetics of the modified GCE was studied using electrochemical impedance spectroscopic (EIS) as seen in Fig. 4c. The frequency range was selected between 1 to 10^5^ Hz, and the initial potential was set to 0.206 V. The values of the components of the circuit are mentioned in Table 1. Lower *R*_ct_ value of composite reveals that better charge transfer takes place with the modified GCE as compared to the bare GCE. The EIS indicated that the conductivity, interface resistance, and electrical stability of Co-BTC-GO-MOFs modified GCE are much superior as compared to others. The semi-circle represents the total impedance of the system. The EIS curve of modified GCE shows a very small radius, which is attributed to fast electron transfer.^23–25^ A remarkable increase in charge-transfer resistance (*R*_ct_) has also been observed from the data with the immobilization step. The increased value of *R*_ct_ further recognized the immune complex formation between the antibody and antigen.

**Table 1:** Values of various components of the Randles equivalent circuit model^a^

Material	*R*_s_ (Ω)	*R*_ct_ (Ω)	*C*_dl_ (F g^−1^)	*W* (Ω s^−1/2^)
GCE	23.1	240.9	4.788 × 10^−7^	0.00026320
Co-BTC-GO/GCE	106.2	46.97	1.852 × 10^−7^	0.00029700
ab-Co-BTC-GO/GCE	114.8	269.5	3.574 × 10^−7^	0.00016360
ag-ab-Co-BTC-GO/GCE	114.6	1466	2.698 × 10^−7^	0.00004964

a Where, *R*_s_: resistance of the solution, *R*_ct_: charge transfer resistance, *C*_dl_: double-layer capacitance and *W*: Warburg impedance.

**Fig. 5:**
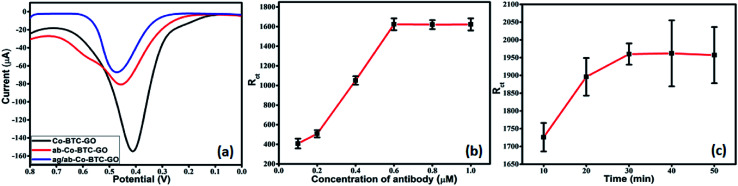
(a) DPV curves of various modified GCEs in 1 mM [Fe(CN)_6_]^3−/4−^ and 0.01 M PBS (pH 7.4) GCE; (b) calibration plot for variation in charge transfer resistance (*R*_ct_) values taken from EIS measurements *vs.* concentration of antibodies in 1 mM [Fe(CN)_6_]^3−/4−^ in 0.01 M PBS of modified GCE with different antibody concentrations; (c) calibration plot between variations in *R*_ct_*vs.* incubation time of antibody modified electrode in BSA.

The experimental conditions were optimized to obtain the optimal electrochemical response during analysis. It is very well-known that the concentration of antibody required for immobilization and BSA incubation time greatly affects the sensitivity, specificity and sensor performance. Therefore, we investigated the concentration of antibody required for immobilization and the BSA incubation time to block all non-specific binding sites effectively. In this study, DPV was performed to analyse the analytical performance and detection capability of the electrochemical immunosensor. The DPV measurements were carried out in 1 mM [Fe(CN)_6_]^3−/4−^ in 0.01 M PBS as the redox couple. The pH value was kept 7.4 as the biological protein get destroy in highly acidic or alkaline conditions. Under a neutral environment, smaller electrostatic repelling is favourable to immune reaction. Each step of fabrication of the sensor electrode was monitored by the electrochemical performance through measurement of DPV. DPV of Co-BTC-GO-MOFs displayed a peak current at the potential of 0.45 V as shown in Fig. 5a. The output current decreases on the attachment of the antibody and further decreases on the formation of antigen/antibody complex. This is due to the non-conducting nature of the antigen and antibody.

Further, the optimization to achieve the most efficient output responses was carried out by varying the concentration of antibody required for immobilization and incubation time in antigen solution. Optimization was performed using EIS. The modified electrode was activated in EDC/NHS and then incubated in varying concentrations of antibody. The charge transfer resistance (*R*_ct_) value of the modified electrode did not increase on increasing the antibody concentration above 0.6 mM (Fig. 5b). Therefore, 0.6 mM was chosen as the optimum concentration for the most efficient immobilization of the antibody. After immobilization, the peak current was decreased, which indicated the successful bonding of the antibody onto the surface of Co-BTC-GO-MOFs/GCE as depicted in Fig. 5a.

Further, EIS measurements were conducted to optimize the BSA incubation time of the electrode. After 30 min, a constant signal with very minimum deviation was observed (Fig. 5c). This depicted that after 30 min was the optimal incubation time of BSA to block the non-specific binding sites effectively and to carry out further studies.

**Fig. 6:**
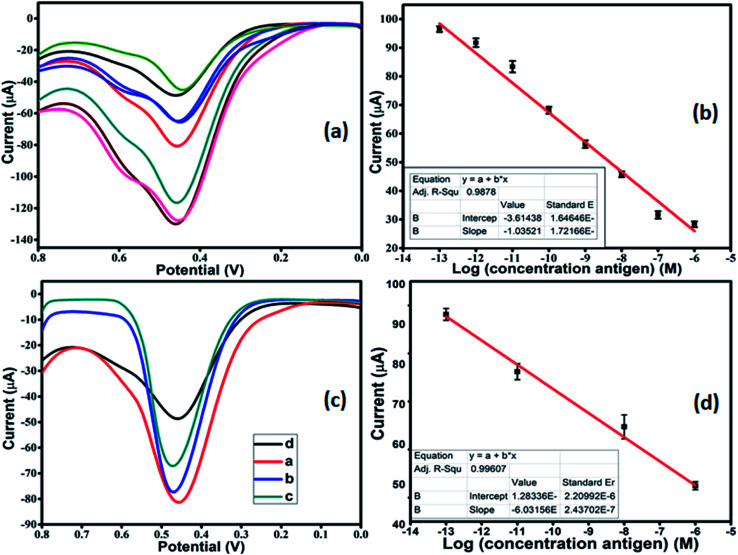
(a) DPV of ab-Co-BTC-GO/GCE modified with varying concentrations of antigen (from 10^−13^ M to 10^−6^ M) in 1 mM [Fe(CN)_6_]^3−/4−^ in 0.01 M PBS; (b) calibration plot between peak current *vs.* log[antigen]; (c) DPV of ab-Co-BTC-GO/GCE with increasing concentration of antigen from a to d in animal serum sample; (d) calibration plot between peak current *vs.* log[antigen] in animal serum.

After optimization, DPV was performed in 1 mM [Fe(CN)_6_]^3−/4−^ in 0.01 M PBS as the redox couple, to carry out the detection of antigen. The modified working electrode was incubated with varying concentrations of antigen from 10^−13^ M to 10^−6^ M. As the concentration of antigen was increased, a clear decrease in the current was observed (Fig. 6a). This can be attributed to the non-conducting nature of the antigen, which leads to a reduction in the current response. The calibration plot was plotted with peak current and antigen concentration in Fig. 6b. The linear regression for the plot came out to by using eqn (ii):

ii *I*_p_ (A) = 3.614 × 10^−5^ + 1.035 × 10^−5^ log[antigen] (M); *R* = 0.98785

The LOD for the fabricated electrode was calculated from the calibration plot, which is found to be 5.23 pM (using relation 3S/N). The present work provided better LOD than the previously reported sensor. The performance comparison of the current sensor with reported sensors in the literature is summarized in Table 2.

**Table 2:** Comparison of other techniques with the proposed sensor for VEGF_165_ determination

Materials	Analytical method	Linear range	Detection limit	References
AgNPs-enhanced time-resolved fluorescence	Fluorescence	0.1 to 16 nM	0.08 nM	26
Aptamer–target recognition	Electrochemiluminescence	1 pM to 20 nM	0.2 pM	27
DNA aptamer	Luminescence	1–20 pg mL^−1^	1 ng mL^−1^	28
Porous poly(ethylene) glycol diacrylate (PEGDA) hydrogel microspheres	ELISA	—	0.9 pg mL^−1^	29
AgVO_3_	Photoelectrochemical	10 fM to 10 nM	3 fM	30
Co-BTC-GO-MOFs	DPV	10^−13^ to 10^−6^ M	5.23 pM	This work

The VEGF plays an important role in the development of various tumors and metastasis. Therefore, to investigate the feasibility and potential analytical applicability of the fabricated sensor, the developed immunosensor was assessed through the recognition of VEGF_165_ in three different serum sample dilutions. The precision of the Co-BTC-GO-MOFs was accessed by adding standard solutions of different concentrations. Fig. 6c depicts the measured concentrations of spiked VEGF-A solution in serum samples according to the linear curve fitting in the range of from 10^−13^ M to 10^−6^ M. The linear regression equation came out from eqn (iii):

iii *I*_p_ (μA) = 1.2833 × 10^−6^ + 6.0135 × 10^−5^ log[antigen] (nM); *R* = 0.99607

From DPV results; it can be predicted that the Co-BTC-GO-MOFs could be a promising tool for determination of VEGF_165_ in the practical serum specimens from the cancer patients as an approach with sensitivity and viability. Therefore, the proposed Co-BTC-GO-MOFs have a great potential for clinic diagnosis of cancer at the early stage for proper treatment and cure of the patient.

The reproducibility of scientific assays is an important parameter that needs to be considered in order to develop a reliable sensor.^31^ Thus, three modified electrodes were independently prepared to evaluate the reproducibility of the proposed Co-BTC-GO-MOFs to assess at the optimized experimental conditions. The obtained results were used to determine the mean relative standard deviation (RSD), where the currents of the obtained DPV signals of the proposed Co-BTC-GO-MOFs based immunosensor was found to be 2.54%. These results clearly indicated that the developed electrodes are highly reproducible. Additionally, the low % RSD values with the Co-BTC-GO-MOFs represents the efficiency of the fabricated electrode for the detection of VEGF_165_.

To investigate the stability of the Co-BTC-GO-MOFs based immunosensor, the modified electrode was kept in PBS with a pH of 7.4 at room temperature under ambient for 1, 3, 6, 10, 14 and 30 days. The response of the stored electrode was 99.8, 99.6, 99.5, 99.03, 98.09 and 98.02%, respectively. The response of the modified electrode showed excellent storage stability.

## 4 Conclusion

In this study, a novel and ultrasensitive immunosensor has been successfully developed for the detection of VEGF_165_ using Co-BTC-GO-MOFs as the biosensing platform. The developed immunosensor exhibits a wider linear range with lower detection limit, better anti-interference as well as higher stability and repeatability to the VEGF_165_ under the optimized experimental parameters. The LOD was found to be 5.23 pM with a linear range of 10^−13^ to 10^−6^ M. This result showed that Co-BTC-GO-MOFs could be applied in point of care scenarios. Collectively, these findings shed light on the modern electrochemical analysis platform, which gives us great potential for portable on-site detection of VEGF_165_, based on this proof-of-concept sensor. Moreover, how these advances can become clinically useful tools. In view of an excellent LOD with a wide linear range, this newly synthesized Co-BTC-GO-MOFs represents an insightful paradigm for effective cancer detection, which could be translated to the clinics.

## Author contribution

Sima Singh – conceptualization, synthesis, characterization, detection and drafting of manuscript; Arshid Numan – conceptualization, electrochemical detection, reviewing and editing; Yiqiang Zhan – fund for the project, supervision, reviewing and editing; Vijender Singh – reviewing of manuscript; Aftab Alam – drafting and reviewing of manuscript; Tran Van Hung – data curation, original draft and editing; Nguyen Dang Nam – supervision, reviewing and final proof read.

## Conflicts of interest

There is no conflict of interest and disclosures associated with the manuscript.

## Supplementary Material
